# An orthotopic mouse model of gastric cancer invasion and metastasis

**DOI:** 10.1038/s41598-017-19025-y

**Published:** 2018-01-16

**Authors:** Rita A. Busuttil, David S. Liu, Natasha Di Costanzo, Jan Schröder, Catherine Mitchell, Alex Boussioutas

**Affiliations:** 1 Upper Gastrointestinal Translational Research Laboratory, Peter MacCallum Cancer Centre, Parkville, VIC Australia; 2 Sir Peter MacCallum Department of Oncology, The University of Melbourne, Parkville, VIC Australia; 3 Department of Medicine, Royal Melbourne Hospital, The University of Melbourne, Parkville, VIC Australia; 4 Cancer Biology and Surgical Oncology Research Laboratory, Peter MacCallum Cancer Centre, Parkville, VIC Australia; 5 Department of Surgery, Austin Hospital, Heidelberg, VIC Australia; 6 Bioinformatics Division, The Walter & Eliza Hall Institute of Medical Research, Parkville, VIC Australia; 7 School of Computing and Information Systems, The University of Melbourne, Parkville, VIC Australia; 8 Department of Pathology, Peter MacCallum Cancer Centre, Parkville, VIC Australia

## Abstract

Gastric cancer is a leading cause of cancer death worldwide, with advanced stage being correlated to the level of tumour invasion and metastasis. Current research is heavily focused on the identification and development of efficacious therapeutics targeting these fundamental hallmarks of cancer, however there are currently no animal models that mimic the invasive phenotypes observed in humans. To address this we have developed an orthotopic mouse model whereby gastric cancer cell lines are tagged with luciferase and injected into the subserosal layer of the stomach. This allows for the monitoring of primary tumour growth and metastasis in real-time as well as quantitation of the degree of tumour invasion through the stomach wall by immunohistochemistry. We have three models based on the degree of invasion and metastasis that are cell line specific: The AGS cells develop into invasive tumours by 4-weeks with no evidence of metastases, MKN45 cells are moderately metastatic with minimal invasion till week 2 and MKN28 cells are highly metastatic and fully invasive by week 1. These models have utility as a tool for testing the efficacy of anti-tumour, anti-invasive and anti-metastatic therapies in the setting of gastric cancer, which currently has poor treatment options.

## Introduction

Gastric cancer (GC) is currently the fifth most common cancer globally and the third highest cause of cancer related deaths worldwide^1^. It is a potentially curable disease with survival being documented at greater than 90% for patients diagnosed at early stage^2^; however this decreases to less than 20%^3^ when diagnosis involves advanced stage disease. Advanced stage disease is directly correlated with the level of invasion of the cancer through the submucosal layer of the stomach and, at more advanced stages, into adjacent structures or distant sites.

Invasion is a fundamental property of cancer^4,5^ and occurs when cancer cells acquire the ability to penetrate the surrounding tissue. Invasion is dependent on the ability of cells to separate from the primary tumour and to breach the muscularis mucosa and extracellular matrix. In gastric cancer the level of invasion is measured by T-stage. Early stage tumours exhibit minimal invasion and lack nodal metastases. Distant invasion, or metastasis, typically occur by hematogeneous or lymphatic spread.

Understanding the molecular mechanisms by which cancer cells spread from the primary tumour is fundamental to the development of effective therapies targeting GC invasion and metastasis. At present, our ability to test any candidate biomarkers or therapeutics in this setting is limited by the lack of available and suitable experimental models. Several *in vitro* model systems have been developed for different cancer types, including GC, however they lack the contribution of host stroma making them useful in the initial testing phases but troublesome in final stages of validation.

The lack of available models which mimic the invasion phenotype seen in humans makes it difficult to test and validate the efficacy of “anti-invasion” therapeutics *in vivo*. Existing models of GC include the gp130 (757 F/F) mouse which develops macroscopic lesions in 100% of mice with a latency period of 6 weeks; however these cancers rarely exhibit an invasive phenotype^6^. A number of recent studies have reported using orthotopic transplant to generate models of GC using cell lines or patient derived tumours. Implantation methods include subserosal injection of cells^7^, subserosal implantation of tumour pieces^8^ as well as attachment of tumour pieces using OB glue^9^ or by stitching^10^. All of these models result in tumour formation at the implantation site and in some cases are associated with local invasion into the liver or distant metastasis to other sites. However, none of these models allow for the detection and quantification of invasion through the muscularis mucosa and into the gastric mucosa.

In this study we describe the generation of stably expressing GFP-luciferase gastric cell lines with varying levels of metastatic potential that are detectable *in vivo* using bioluminescence. Using these cells, and an orthotopic transplantation model we were able to detect and visualise growth of the primary tumour as well as track local invasion and metastasis in real time. This novel model will be useful for studying the biological effects of invasion and metastasis of gastric cancer as well as providing a tool for testing the efficacy of treatments and therapies.

## Methods

### Mice

Bl6/Rag2/GammaC double knockout mice harboring recombinase activating gene-2 (RAG2) and cytokine receptor gamma-chain (gammaC) mutations were bred and maintained in-house under specific pathogen-free conditions in the research facility of the Peter MacCallum Cancer Centre. Animals were housed in an IVC Optimice caging system on corn cob bedding and were maintained on a 12 hour light/dark cycle at constant temperature. All interventions were performed during the light cycle on both male and female mice. All animals had free access to water and food (standard chow). Methods were carried out in accordance with relevant guidelines. All experimental protocols were approved by the Institutional Animal Care and Use Committee at the Peter MacCallum Cancer Centre (E537).

### Cell culture

The human gastric cancer cell lines MKN45, AGS and MKN28 were a kind gift from Professor Andy Giraud (Murdoch Childrens Research Institute). MKN-45 and AGS were cultured in DMEM and MKN-28 cells were cultured in RPMI. In all cases media was supplemented with 10% (w/v) fetal bovine serum, penicillin (100 U/ml) and streptomycin (100 ug/ml) (Invitrogen, Carlsbad, CA) and were maintained at 37 °C in a humid incubator with 5% CO_2_. The medium was replaced three times weekly, and cells were serially passaged using 0.1% trypsin. Cell line identity was verified using STR analysis (outsourced to The Gandel Charitable Trust Sequencing Centre) using the PowerPlex HS16 system kit and cross validated against ATCC and DSMZ databases.

### Cell lines

Well characterised commercially available cell lines AGS, MKN-45 and MKN-28 were selected based on molecular profiles^11^, representation of the major TCGA molecular subtypes^12^ and from their tissue of origin (primary-tumour, liver-metastasis and lymph-node metastasis, respectively) (Supplementary Fig. 1a–c). Molecular data for the cell lines generated by the Cancer Cell Line Encyclopedia (CCLE)^13^ was extracted using cBIOPortal^14,15^ and COSMIC^16^ databases and TCGA subtype was inferred using this information as well as other published data (Supplementary Fig. 1d).

The AGS cell line was derived from the primary gastric cancer of a 54 year old female which exhibited characteristics of both Lauren subtypes^17^. This cell line was previously reported to be microsatellite stable (MSS)^18^, EBV negative^19^ and is neither hyper- or hypo-methylated^16^. AGS cells have limited copy number changes but a large number of SNV’s (Supplementary Fig. 1a & d) many in genes which are commonly found to be mutated in GC including *CDH1*, *PIK3CA*, *RHOA*, *KRAS* and *CTNNB1*. These characteristics suggest that the AGS cell line most resembles the TCGA^12^ genomically stable (GS) subtype.

The MKN45 cell line was derived from the liver metastasis of a 62 year old patient with a poorly differentiated primary gastric cancer of diffuse histology and exhibits a deranged copy number profile and a large number of genes with gains and losses and very few SNV’s, none of which occurred in genes commonly mutated in GC (Supplementary Fig. 1b & d). This cell line is also MSS^20^, EBV negative^19^ and has no evidence of methylation^16^ suggesting that it is most likely to be of the TCGA CIN subtype^12^.

The MKN-28 cell line was derived from a lymph node metastasis of a 70 year old female with a well differentiated primary gastric cancer of intestinal histology. This cell line also has had moderate copy number gains and losses as well as a moderate number of genes with SNVs, including *TP53* (Supplementary Fig. 1c & d). This cell line has been reported as MSI-L^18^, EBV negative^19^ and non-methylated^16^ and based on these characteristics most closely resembles the TCGA CIN subtype^12^.

### Preparation of luciferase virus and infection of target cells

Luciferase and eGFP were stably expressed in cell lines using the the pFUGW lentiviral vector provided by Dr Mark Shackleton (Peter MacCallum Cancer Centre)^21^. Lentiviral particles were produced using Lenti-X packaging vectors into HEK293T cells. Viral particles were filtered through a 0.45 µm filter and concentrated using an Amicon Ultra-15 Centrifugal Filtration Unit (Merck Millipore, Bayswater, Australia) and stored at −80C until required. Target cells (MKN-45, AGS and MKN-28) were seeded in 6-well plates and transduced when 60% confluent. After 3 days, cells were FACS sorted (BD FACSAria Fusion) to identify eGFP positive populations. eGFP positive cells were reseeded and expanded prior to injection into mice. Prior to injection sub-confluent cell cultures at >90% viability were harvested using trypsin-EDTA (Invitrogen) and washed once in medium.

### Intra gastric injection

**Figure 1:**
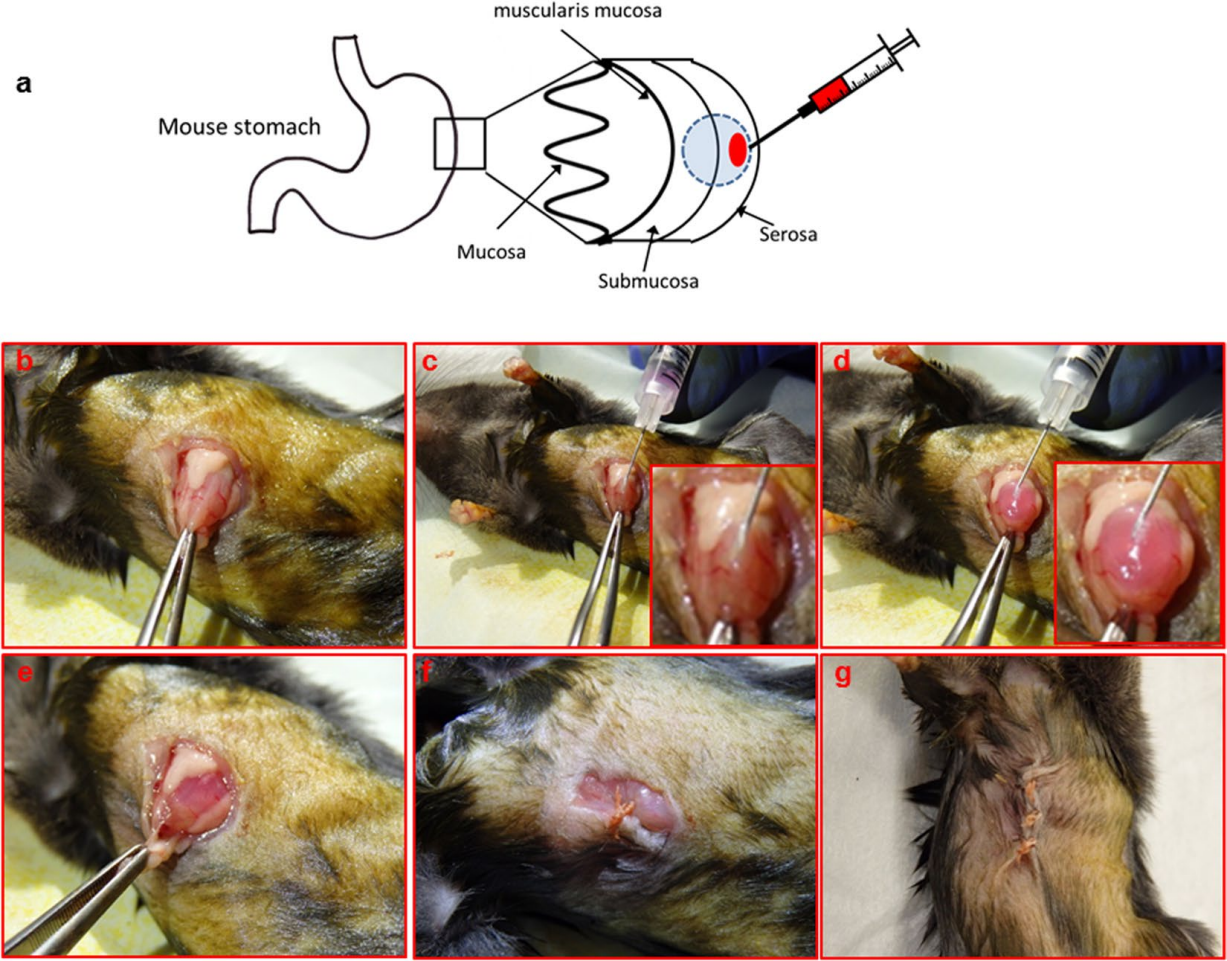
Intra-gastric injection procedure. (**a)** Schematic representation of the intra-gastric injection method. **(b)** Mice were anaesthetised and placed in a dorsal recumbency position under a dissecting microscope. A 5–10 mm midline incision was then made in the skin overlying the mid abdomen. Forceps and surgical scissors were used to exteriorise the stomach. **(c)** A 29 G U-100 insulin syringe containing luciferase-tagged cells (500,000) in matrigel (50 ul) was inserted into the serous side of the stomach with the bevel facing up (see inset). **(d)** With the aid of an assistant the needle was slowly depressed and a matrigel bleb was visible (inset). **(e)** The stomach is carefully returned to the abdominal cavity and **(f)** absorbable sutures were used in a 2 step closure process; first the peritoneal layers were closed **(g)** followed by the skin incision.

This methodology is described in detail in the results section. Briefly, mice were anaesthetised using IP injection of Ketamine/Xylazine and placed in a dorsal recumbency position. Fur around the surgical area was clipped and the area was swabbed with betadine. A 5–10 mm incision was made in the skin overlying the abdomen. Forceps were used to exteriorise the stomach. GFP-luciferase tagged cells (0.5 × 10^6^) in matrigel (50 µl) were injected into the serous side of the stomach (Fig. 1a) and the incision closed using sutures.

### *In vivo* bioluminescence imaging (BLI)

To visualize firefly luciferase, D-Luciferin (Xenogen) was injected IP at 150 mg/kg and mice were allowed to move freely to aid in distribution of luciferin. After 5 minutes mice were anaesthetised using isofluorane before transfer into the imaging chamber of a Lumina II instrument and placed into nose cones. Using IVIS Living Image 3.0 software, images were taken using a field of view of 12.5 cm with binning medium, f/stop 1 and open filter setting. Regions of interest (ROI) were drawn over the upper abdominal area with the intensity of BLI signal quantified in tumour bearing mice by subtracting from an equivalent area in non-tumour bearing mice. Bioluminescent intensity was presented as average radiance (photons/sec/cm^2^/sr). To monitor tumour growth and invasion, mice were typically imaged weekly and weighed twice weekly. Mice were culled at indicated timepoints and autopsies performed immediately. After macroscopic examination the stomach and major organs were removed and imaged. A positive BLI signal indicated presence of tumour or metastasis.

### Immunohistochemistry

Affected tissues were fixed in 10% formalin and embedded in paraffin. Stomachs were cut in half and embedded in both flat and perpendicular orientations. 4 um-sections were used for histopathology. H&E staining was performed and the presence of tumour cells was confirmed by a pathologist. In order to confirm the tumours were of human origin tissue sections were stained with an anti-human mitochondrial stain. Briefly, samples were deparaffinised and antigen retrieval was performed using high pH buffer (Dako North America, Carpinteria, CA). Sections were treated in with 0.05% TBST for 5 minutes and then in Blocking solution (Dako) to block endogenous peroxidase activity. Sections were then blocked using 10% BSA in 0.05% TBST and then incubated with monoclonal mouse anti-human mitochondria antibody (1:500, Millipore) for 1 hour. Sections were then incubated with a biotinylated anti-mouse polymer secondary antibody. The secondary antibody was detected using 3,3′-diaminobenzidine (Dako) and counterstained with Meyer’s hematoxylin. Negative controls were generated by omitting the primary antibody.

### Quantitation of invasion

Sections of the stomach were examined for presence of tumour and scored based on level of invasion. A score of 0 indicated that tumour growth was restricted to the submucosa. Invasion was deemed to have occurred if the tumour had invaded into (score 1), or beyond (score 2) the muscularis mucosa (Supplementary Figures 3 & 4).

Metastatic spread was identified as described above using BLI and confirmed with immunohistochemistry. These were classified further as abdominal (liver, kidney, spleen, mesentery) or thoracic (lung) metastasis. Brain tumours were observed in one case.

### Data availability

The datasets analysed during the current study are available from the Cancer cell line Encyclopedia (CCLE)^13^ http://cancer.sanger.uk/cell_lines and was extracted using cBIOPortal^14,15^ and COSMIC^16^ databases.

## Results

### Intragastric injection of gastric cancer cell lines

To establish an invasive gastric cancer xenograft model which demonstrates invasion through the gastric wall both at local and distant sites, we utilised Rag2/GammaC double knockout mice. These mice contain recombinase activating gene-2 (RAG2) and cytokine receptor gamma chain (gamma C) mutations. They are severely immunocompromised and lack T-, B- and NK-cells^22^. Unlike NOD-SCID mice they show no spontaneous tumour formation and have normal hematopoietic function^22^.

GFP/luciferase positive cell lines were generated as described in the materials and methods. On the day of injection cells were trypsinised into a single cell suspension and washed in DMEM. For each mouse 0.5 × 10^6^ cells were resuspended in 50 ul matrigel and aspirated into pre-chilled 29 G U-100 insulin syringes taking care to avoid bubble formation. Other syringe types and sizes were tested however their size was found to be cumbersome making it difficult to control the depth of injection. Insulin syringes also have the advantage of having no dead space to minimise differences in cell numbers between animals. The syringe containing the matrigel/cell mix was stored on ice until required to prevent setting of the matrigel.

10–12 week old Rag2/GammaC double knockout mice were anaesthetised by intraperitoneal administration of Ketamine/Xylazine at a dose of 100 mg/kg and 20 mg/kg respectively. The surgical site was subsequently shaved and swabbed with 70% alcoholic chlorhexidine (0.5%). With the mouse in a supine position, a 10 mm sub-xiphoid midline incision was made. The stomach was then exteriorised using forceps (Fig. 1b). A dissecting microscope was used to guide the needle (bevel side up) containing 50ul of the cell/matrigel suspension into the subserosal layer of the antral region of the stomach (Fig. 1c and inset). The injection was performed slowly to prevent leakage due to excess pressure. The needle was withdrawn after 20 sec to allow the matrigel to set and prevent inadvertent abdominal seeding of the tumour cells. Successful positioning of the transplant was confirmed by the presence of a matrigel “bleb” (Fig. 1d). In our experience this step was best performed with the aid of an assistant for accuracy.

The stomach was carefully returned to the abdominal cavity (Fig. 1e) followed by closure of the peritoneum and skin with absorbable 4.0 and 3.0 vicryl sutures respectively (Fig. 1f & g). Mice were subsequently returned to a clean box on a heat pad to recover overnight and were monitored after 2 hours and again the following morning. Operative mortality was only observed in 1 instance and was related to incompatibility with anaesthetic.

### Tumour growth kinetics

Mice were monitored and weighed twice weekly and bioluminescence imaging was performed weekly where possible. In all cell lines tested the weight of mice which ultimately developed tumours was consistent with those which did not develop tumours (Supplementary Figure 2) suggesting that tumours were not obstructing the stomach or bowel. Monitoring of the mice by BLI commenced 7 days after implantation of the tumour cells and was repeated weekly for the duration of the experiment. The Xenogen IVIS imaging system was used to perform a whole body scan and evaluated the average radiance (photons/second/cm^2^/radian). A reading above background, determined by measuring a corresponding region from a non-injected mouse, was considered positive (BLI+).

**Figure 2:**
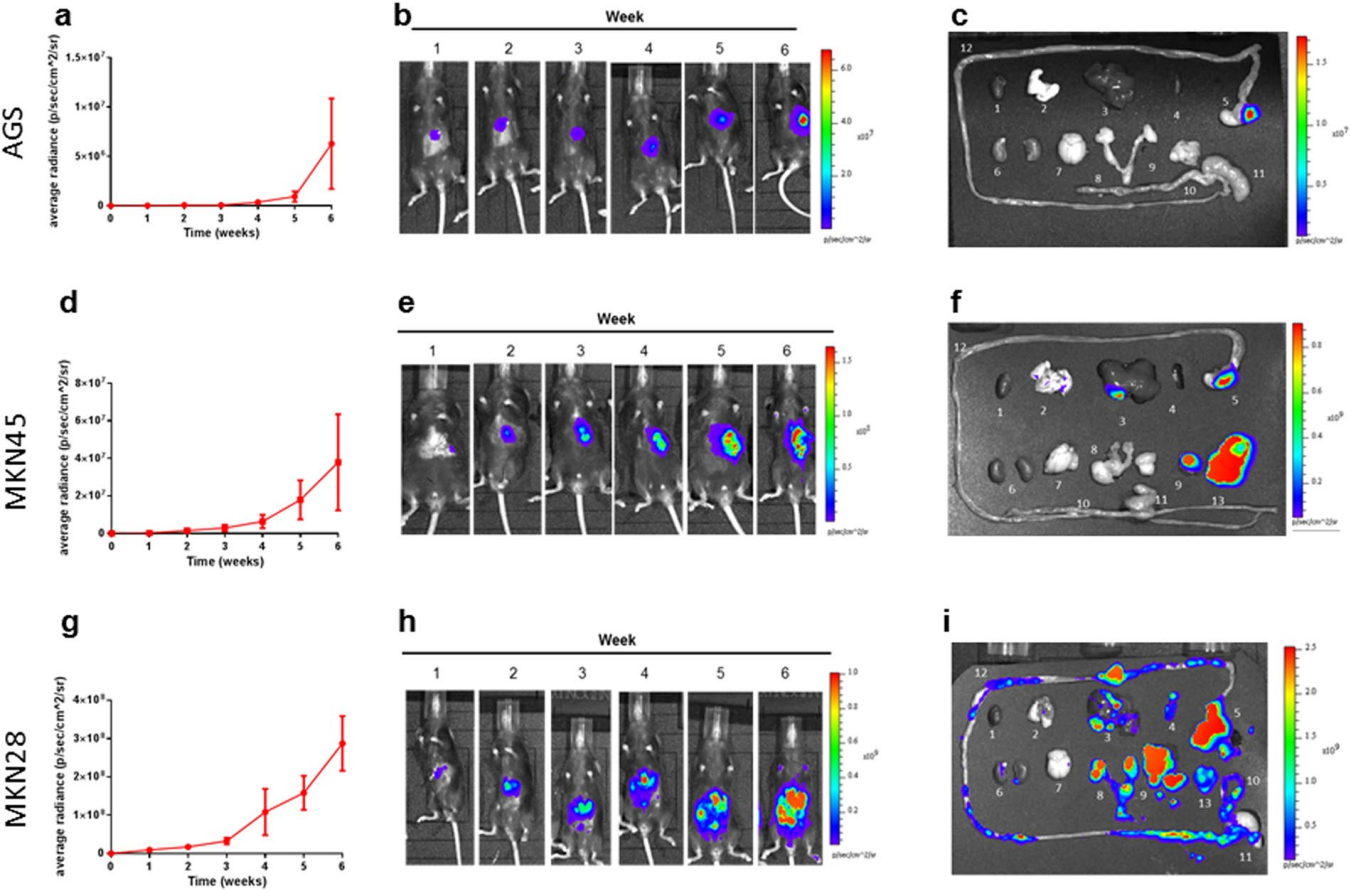
Experimental profile of mice injected with AGS, MKN45 and MKN28 cells. **(a,d,g)** Shows an increase in average radiance in mice which developed tumors (over the 6-week experimental period). **(b,e,h)** Representative images of weekly BLI scanning. **(c,f,i)** After the final scan mice were euthanized, organs and tissues were removed and imaged *ex vivo*. Color bar represents light-intensity levels as average radiance (photons/sec/cm^2^/sr). (1-heart; 2- lung; 3-liver; 4-spleen; 5-stomach; 6-kidney; 7-brain; 8-sex-organs; 9-mesentery; 10-large-intestine; 11-caecum; 12-small-intestine; 13-peritoneum).

Tumour uptake with AGS cells was 72.4% (Supplementary Table 1) determined by positive bioluminescence signal at week-2. Similar injection methodologies previously reported a lack of take-rate with this cell line when SCID mice were used^7^. Average radiance increased over the experimental period from 1.85 × 10^4^ photons/second/cm^2^/radian at week-1 to 6.28 × 10^6^ photons/second/cm^2^/radian at week-6 (Fig. 2a & b). Mice were culled at 6-weeks, immediately following the corresponding BLI scan and major tissues/organs imaged *ex vivo*. AGS cells showed tumour growth at the primary injection site (consistent with their origin as a primary GC) but no metastases by 6-weeks (Fig. 2c).

Injection with MKN45 cells resulted in a tumour uptake of 75% (Supplementary Table 1) measured 2-weeks post-injection. Average radiance increased over 6-weeks from 2.1 × 10^5^ to 3.79 × 10^7^ photons/second/cm^2^/radian (Fig. 2d & e). Metastases were observed in 100% of mice at 6-weeks post injection predominantly in liver, lung and mesentery (Fig. 2f).

MKN28 cells had the highest tumour uptake of 82% at week-2. (Supplementary Table 1). Tumour burden and BLI signal increased from 9.28 × 10^6^ to 2.87 × 10^8^ photons/second/cm^2^/radian (Fig. 2g & h). Average radiance at 6-weeks was 10- and 100- fold higher than the MKN45 and AGS cells respectively. This was reflected by widespread metastases observed by 6-weeks (Fig. 2i).

### Invasive capability varies among cell lines

**Figure 3:**
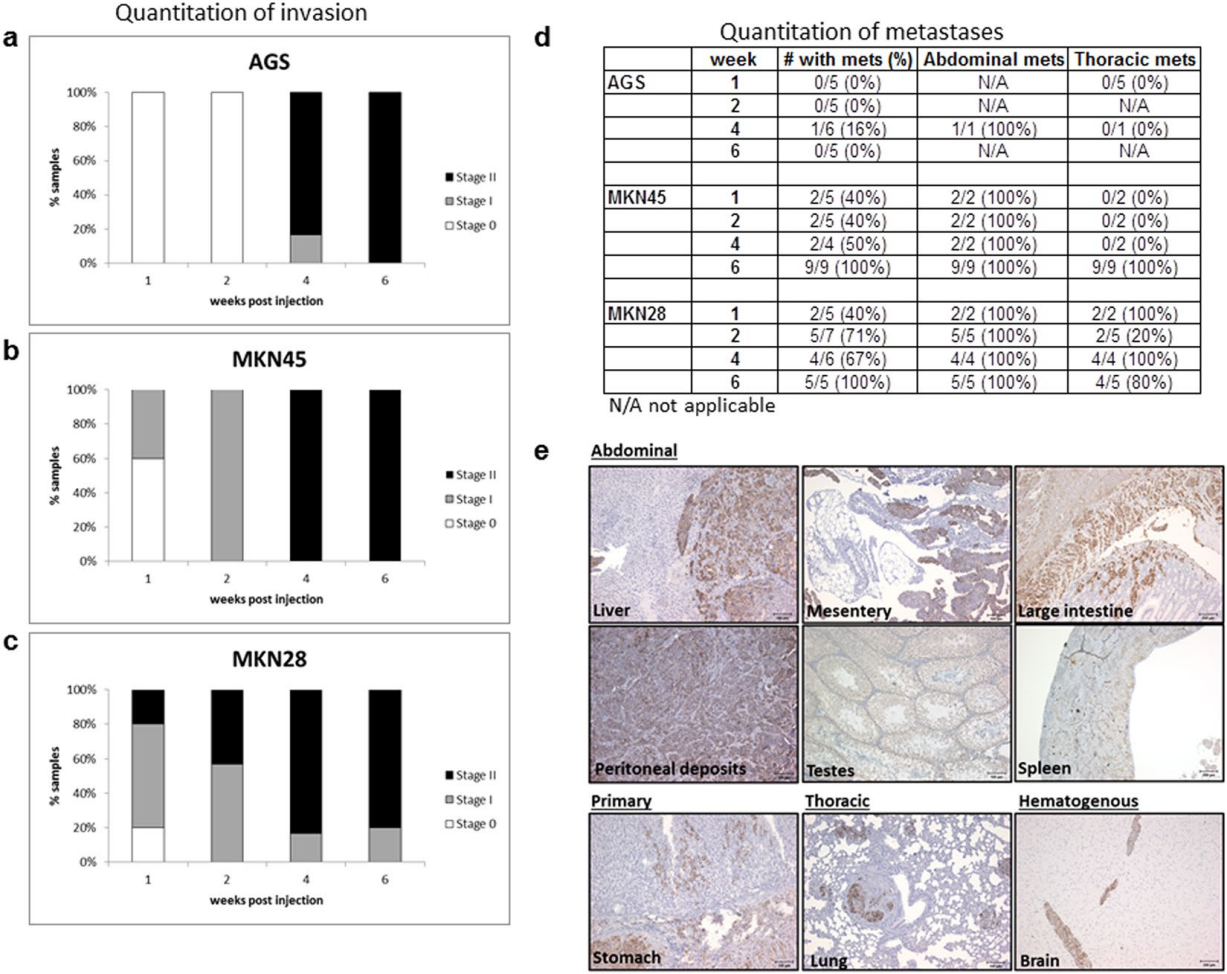
Quantitation of metastatic and invasive capabilities. To quantitate the extent of invasion stomach sections were stained with an anti-human mitochondrial antibody to identify tumor cells and scored based on extent of invasion. Tumors restricted to the submucosa were scored Stage 0. Invasive tumors that had breached and invaded into the muscularis mucosa or beyond were scored Stage I and II respectively (Supplementary Figs 3 & 4). The percentage of mice represented by these invasion scores are shown for **(a)** AGS, **(b)** MKN45 and **(c)** MKN28 cells. **(d)** Presence of metastases was validated by IHC and classified as abdominal or thoracic. **(e)** Representative images of anti-human mitochondrial staining on primary tumour and metastastatic sites from a mouse injected with the MKN28 cell-line at week-6. Scale bar 100 uM.

To determine the kinetics of invasion, mice were injected and subsequently euthanized at 1, 2, 4 and 6-weeks post-injection. Stomachs of BLI+ mice were processed as described in the Supplementary Methods and histological analysis of tissue used to further characterise the spread of invasion. H&E stains enabled anatomical analysis, whilst immunohistochemistry using an anti-human mitochondrial antibody confirmed human origin of the tumours. Invasion was quantitated as in Supplementary Methods and Supplementary Figs 3 & 4. Although the intention was to inject serosally, this layer is extremely thin and even with the assistance of a stereo-microscope submucosal injection could not be excluded and was therefore considered as baseline. Hence, reverse invasion through the muscularis mucosa (stage I) and subsequently mucosa (stage II) is the measure of invasion in this model. When mice were injected with AGS cells tumours were not invasive until week-4 (Fig. 3a) whereas with the MKN45 cell line 40% were invasive at week 1 and 100% were invasive at week-2 (Fig. 3b). MKN28 cells were extremely invasive and metastatic with 100% of tumours breaching the muscularis mucosa as early as 1-week post injection (Fig. 3c). Inadvertent injection of cells into deeper layers or tumour cell leakage during injection of the stomach was ruled out by examining the levels of invasion and metastasis at the early time points (1-week post-injection) in the least invasive cell line, AGS. No invasion beyond the mucosa or metastases was observed.

### Frequency and distribution of metastases is dependent on cell type

The frequency and location of metastases was determined by *ex vivo* imaging of individual organs/tissues which were then confirmed by IHC and described in Fig. 3d and Supplementary Table 2. Metastases were described as being thoracic if detected above the diaphragm (ie. lung) or abdominal if located below the diaphragm (ie. liver, intestines, spleen and sex organs). Metastases found in the brain were classified as hematogenous.

Throughout the 6-week experimental period AGS cells were found to be primarily non-metastatic, however when mice were analysed at a later time point (9-weeks) 80% had developed locally metastatic lesions restricted to the abdominal region (data not shown). MKN45 cells were found to be moderately metastatic (oligo-metastatic model) with 50% of mice showing metastatic lesions, primarily to the liver, by 4-weeks post-injection. By 6-weeks post injection 100% of mice harboured metastatic burden in both abdominal (primarily to liver and caecum) and thoracic (lung) compartments (Fig. 3e). We observed a high rate of metastasis in mice injected with the MKN28 cell line (highly metastatic model). Metastases were observed as early as 1-week post injection (in mesentery and lung) and were detected in 70% of mice by 2-weeks in multiple sites within abdominal and thoracic regions. By 6 weeks metastases were widespread and detected in most tissues and organs (see Fig. 2i for an example). In one case a brain metastases was also detected (Fig. 3e).

## Discussion

The outcome of patients diagnosed with GC is greatly dependent on the stage of disease at diagnosis, with the depth of tumour invasion and degree of nodal involvement being key prognostic factors^23^. Patients with early stage disease who undergo surgery are likely to benefit by long term survival^24^, whilst those diagnosed at more advanced stage with invasive tumours are more likely to harbour unresectable tumours and/or metastatic lesions. From a clinical perspective there is a significant need to improve survival from this disease and potential methodologies include the development of novel therapeutics targeting invasion or metastasis, which compliment current therapeutic strategies.

Although invasion in our model occurs in the reverse direction (serosal to luminal) breach of the basement membrane is still a clinically relevant characteristic. Whilst tumour penetrance was not 100%, the ability to track tumour development in real time using BLI makes it possible to identify those mice in which implantation was unsuccessful early in the experiment.

Given the internal nature of the tumours resulting from this procedure, monitoring of mice could be challenging without the use of BLI. Other groups performing similar studies have used parallel subcutanenous injection of the same number of cells as a surrogate of tumour growth^7^. We have observed that subcutaneous tumours grow at different rates than the intra-gastric tumours suggesting that this is not an appropriate means of animal monitoring. Tracking of animal weight was also uninformative. Our study shows that mouse weight did not differ between tumour bearing and non-bearing mice even once tumours and metastasis were well established. During the experimental duration, mice appeared healthy and active with no evidence of cachexia and lethargy which was observed in other studies^25^. Due to extensive tumour infiltration into the stomach it was not possible to determine tumour weight even at the time of autopsy.

For subsequent testing of anti-invasive therapeutics the ideal time to begin intervention would be once the tumour is established but not yet invading. Based on the data reported here we would recommend treating mice injected with AGS cells at 1 week post implantation provided that tumour establishment has been confirmed by BLI. For mice injected with the MKN-45 cell line treatment at 1 week post injection would also be appropriate. Due to the extremely aggressive and early invasion seen in the MKN28 cells this line would not be suitable to test the efficacy of such therapeutics.

The stark differences in metastatic burden conferred by these cell lines suggests that model would also be suitable for the testing of therapies which target, or aim to prevent metastasis. In this instance the moderately metastatic MKN-45 cell line or the highly metastatic MKN-28 cell line would be the most appropriate. Similarly this model would also be useful for the testing of new therapies targeting primary gastric cancer.

The use of cell lines is a limitation in the system but can also be a strength, in that, the molecular characteristics of the cell lines are well recognised and homogenous. Given primary GC is molecularly heterogeneous this model system can be used to examine specific subsets of molecular derangements by studying homogenous populations of tumours using specific cell lines. In the future this technique could be used to engraft human PDX (patient derived xenografts) in a similar way, to examine their growth and invasion. This may need to be done after expansion of primary tumours in existing PDX models. The resulting tumours would then be homogenised into single cells which can then be tagged using the same luciferase vector^21^. Here we have utilised cell lines which represent the genomically stable (GS; AGS) and chromosomal unstable ((CIN; MKN45 and MKN28) subtypes described by TCGA molecular profiling of human GC, which may help us to understand the molecular characteristics of these subtypes *in vivo*.

We believe that our novel orthotopic model is clinically relevant and recapitulates the processes of gastric cancer invasion and metastasis. This model can be applied to test anti-invasive and anti-metastatic therapeutics, as well as to help elucidate the biological processes of primary tumour growth, underlying invasion and metastasis of gastric cancer.

## Electronic supplementary material

Supplementary information
