# Supplementary material for: An orthotopic mouse model of gastric cancer invasion and metastasis
Source: Sci Rep. 2018 Jan 16;8:825. doi: 10.1038/s41598-017-19025-y (PMC5770387; doi:10.1038/s41598-017-19025-y)
Supplement: Supplementary file 1 — Supplementary information [file 41598_2017_19025_MOESM1_ESM.pdf]

Supplementary files:

An orthotopic mouse model of gastric cancer invasion and metastasis

Rita A. Busuttil, David S. Liu, Natasha Di Costanzo, Jan Schröder, Catherine Mitchell and Alex Boussioutas

a AGS b MKN45 c MKN28

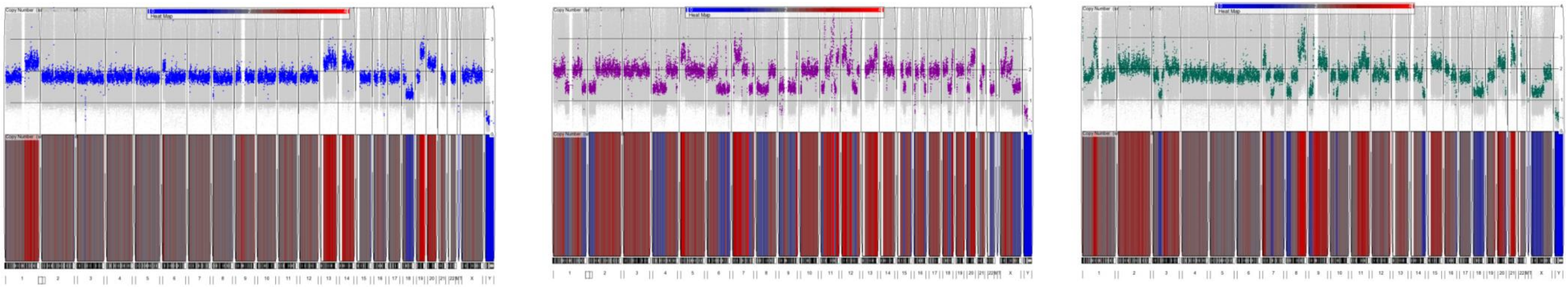

d

cell line	source	Lauren subtype	key mutated genes	MSI status	EBV status	TCGA subtype
AGS	primary gastric	Mixed	CDH1 PIK3CA RHOA KRAS CTNNB1	MSS	neg	GS
MKN45	liver metastasis	DGC		MSS	neg	CIN
MKN28	lymph node metastasis	IGC	TP53	MSI-L	neg	CIN

Supplementary Figure 1: Molecular profiles of the three cell lines tested. CNV plots showing the genomic landscape of the cell lines (a) AGS, (b) MKN45 and (c) MKN28. Data was obtained from [11] (http://cancer.sanger.ac.uk/cell_lines) (d) Cell line characteristics based on origin, histology and TCGA subtype.

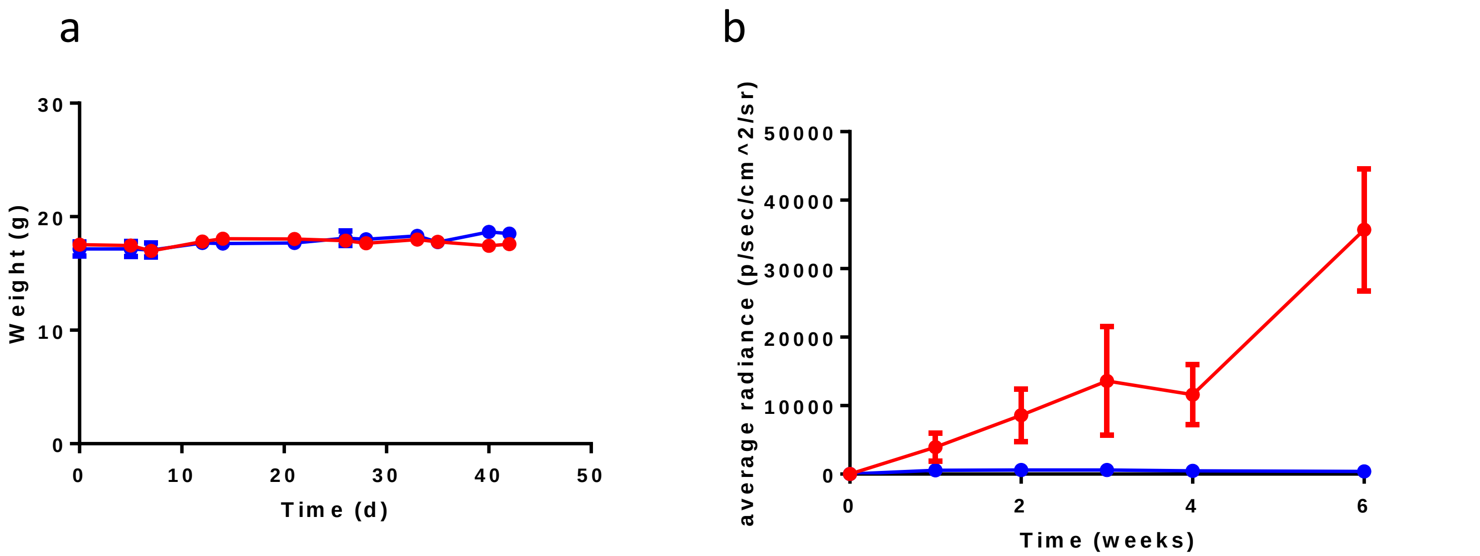

Supplementary Figure 2: Comparison of mouse weight and BLI signal in tumour bearing and non-bearing mice. (a) Monitoring of weight was not a suitable surrogate for identifying mice which ultimately developed tumours (blue circles; n=9) from those which did not (red circles; n=3). This was in contrast to the BLI measurements taken from the same mice **(b)** showing an increase in signal which was correlated with tumour growth.

Supplementary Table 1: Take rate following intragastric injection

Cell line	BLI signal at Week 1	BLI signal at week 2
**AGS**	22/38 (57.9%)	21/29 (72.4%)
**MKN45**	26/33 (78.8%)	21/28 (75%)
**MKN28**	23/32 (71.9%)	18/22 (82%)

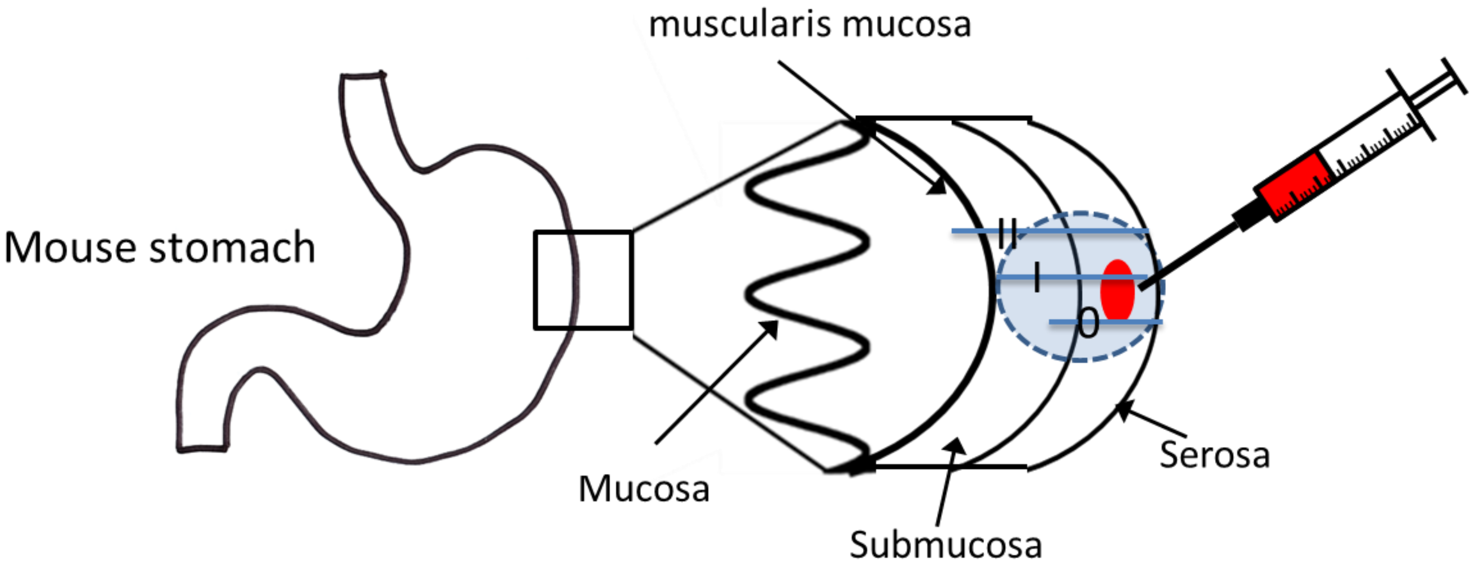

Supplementary Figure 3: Quantitation of gastric cancer invasion. At the end of the experimental period stomachs were imaged and BLI signal confirmed before collection in formalin. The stomach was cut in half and embedded in flat and perpendicular orientations before being embedded in paraffin (FFPE). FFPE sections of the stomach were stained with an anti-human mitochondrial antibody and H&E to identify tumour cells. Tissue was examined for the presence of tumour and scored based on the degree of invasion. A score of 0 indicated that tumour growth was restricted to the submucosal area and was not invasive. Penetration of the tumour into the muscularis mucosa (Stage I) or beyond (Stage II) were positive indicators of invasion. Both orientations were graded and the extreme measurement was taken.

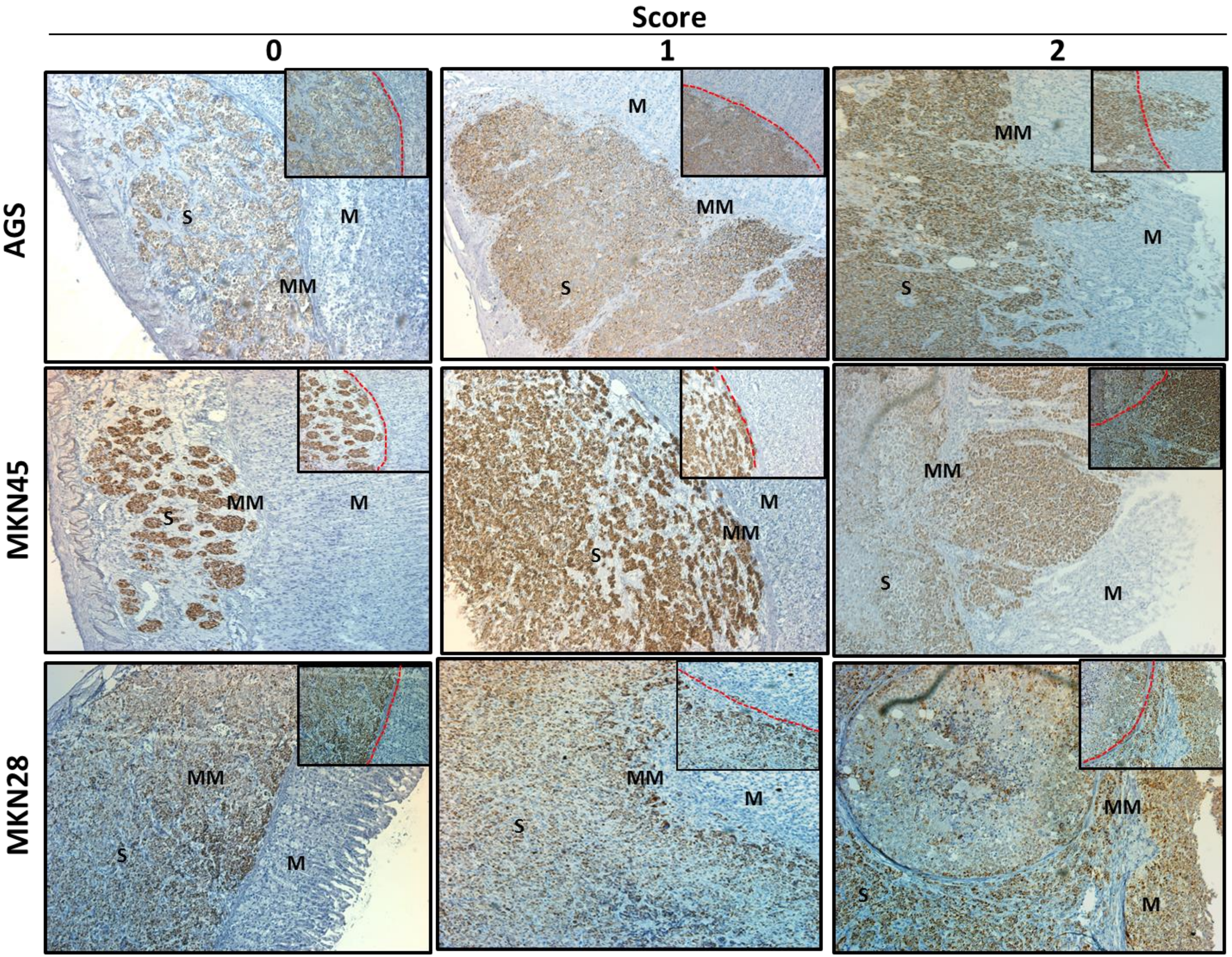

Supplementary Figure 4: Representative images of invasion scoring. Images of stomachs stained with anti-human mitochondrial stain representing invasion scores of 0, 1 and 2 are shown for the three cell lines studied (AGS, MKN45 and MKN28) indicating the locations of the submucosa (S), mucosa (M) and the muscularis mucosa (MM). (Magnification X10). Inset images (x20) indicate the edge of the muscularis mucosa (red line).

Supplementary Table 2. Details of metastatic sites

				Abdominal										Thoracic	hematogenous
	week	mouse	Any mets?	*heart*	*liver*	*spleen*	*kidney*	*sex organs*	*mesentery*	*large intestine*	*caecum*	*small intestine*	*peritoneum*	*lung*	*brain*
AGS	1	1	N												
		2	N												
		3	N												
		4	N												
		5	N												
	2	1	N												
		2	N												
		3	N												
		4	N												
		5	N												
	4	1	N												
		2	N												
		3	N												
		4	N												
		5	N												
		6	Y						Y						
	6	1	N												
		2	N												
		3	N												
		4	N												
		5	N												
MKN45	1	1	Y					Y							
		2	N												
		3	N												
		4	N												
		5	Y				Y								
	2	1	Y		Y						Y				
		2	N												
		3	Y						Y						
		4	N												
		5	N												
	4	1	N												
		2	N												
		3	Y		Y	Y		Y							
		4	Y		Y				Y						
	6	1	Y		Y									Y	
		2	Y		Y			Y			Y		Y	Y	
		3	Y		Y			Y			Y		Y	Y	
		4	Y		Y						Y		Y	Y	
		5	Y		Y			Y			Y			Y	
		6	Y		Y			Y			Y		Y	Y	
		7	Y		Y			Y					Y	Y	
		8	Y		Y			Y			Y			Y	
		9	Y		Y			Y			Y			Y	

**MKN28**	1	1	Y					Y	Y					Y	
		2	Y						Y					Y	
		3	N												
		4	N												
		5	N												
	2	1	Y			Y			Y		Y			Y	
		2	Y		Y			Y	Y		Y			Y	
		3	Y					Y							
		4	Y					Y							
		5	Y						Y						
		6	N												
		7	N												
	4	1	N												
		2	Y		Y			Y	Y		Y			Y	
		3	N												
		4	Y	Y	Y	Y	Y	Y	Y	Y	Y	Y	Y	Y	
		5	Y		Y	Y	Y	Y	Y	Y	Y	Y	Y	Y	
		6	Y		Y	Y	Y	Y	Y	Y	Y	Y	Y	Y	
	6	1	Y	Y	Y	Y	Y	Y	Y	Y	Y	Y	Y	Y	
		2	Y	Y	Y	Y	Y	Y	Y	Y	Y	Y	Y	Y	
		3	Y				Y	Y				Y			
		4	Y	Y	Y	Y	Y	Y	Y	Y	Y	Y	Y	Y	
		5	Y		Y	Y	Y	Y	Y	Y	Y	Y	Y	Y	Y

Y= yes; N=no
